# IQOS point-of-sale marketing: a comparison between Arab and Jewish neighborhoods in Israel

**DOI:** 10.1186/s13584-024-00626-8

**Published:** 2024-08-16

**Authors:** Amal Khayat, Hagai Levine, Carla J. Berg, Lorien C. Abroms, Zongshuan Duan, Yan Wang, Cassidy R. LoParco, Daniel Elbaz, Yuxian Cui, Yael Bar-Zeev

**Affiliations:** 1 Braun School of Public Health and Community Medicine, Faculty of Medicine, The Hebrew University of Jerusalem – Hadassah Medical Center, Ein Kerem, PO Box 12272, 911200 Jerusalem, Israel; 2 Milken Institute School of Public Health, George Washington University, Washington, DC USA; 3 Department of Population Health Sciences, School of Public Health, Georgia State University, Atlanta, GA USA

**Keywords:** IQOS, Heated tobacco products, Point-of-sale, Marketing

## Abstract

**Background:**

Philip Morris International’s IQOS, with its heatsticks (HEETS), is the heated tobacco product with the largest global market share. IQOS and/or electronic cigarettes use rate is higher among Arabs vs. Jews in Israel. This paper aims to compare IQOS point-of-sale marketing strategies, and regulatory compliance in Arab vs. Jewish neighborhoods in Israel.

**Methods:**

We integrated data from two separate studies including a cross-sectional survey with IQOS retailers (December 2020–April 2021) and audits of points-of-sale that sold IQOS/HEETS (April 2021–July 2021) in 5 large cities in Israel, after marketing restrictions including a points-of-sale display ban and plain packaging became effective in Israel (January 2020). The survey included 69 points-of-sale (21 Arab, 48 Jewish neighborhoods) and the audits included 129 points-of-sale (48 Arab, 81 Jewish neighborhoods). Comparisons of IQOS marketing strategies between points-of-sale in Arab and Jewish neighborhoods were conducted using Chi-Square test, Fisher’s exact test or Mann–Whitney test, as appropriate. Thematic analysis was used to analyze open-ended questions.

**Results:**

The survey showed that most marketing strategies, such as promotions to customers, were uniform across points-of-sale in Arab and Jewish neighborhoods. The most noteworthy differences were that a higher proportion of retailers from Arab neighborhoods were invited to IQOS parties (47.6% vs. 21.7%, *p* < 0.05) and reported personal communication with a Philip Morris International’s representative (80.0% vs. 51.2%, *p* < 0.05). Additionally, Philip Morris International’s representatives assisted points-of-sale in both Arab and Jewish neighborhoods in implementing the display ban by providing free compliant cabinets and product placement instructions, and directly interacted with customers. The audits showed that points-of-sale in Arab neighborhoods were more compliant with the display ban (25.5% vs. 8.8%, *p* < 0.05), but less compliant with plain packaging (62.5% vs. 79.3%, *p* < 0.05).

**Conclusions:**

There were not many notable differences in IQOS marketing across points-of-sale in Arab vs. Jewish neighborhoods, but Philip Morris International utilized marketing elements of cultural significance, especially for points-of-sale in Arab neighborhoods, such as more personal communication and invitation to social events. Continuous surveillance of tobacco points-of-sale marketing and legislation compliance is needed, with a special focus on demographic/location-based differences.

## Background

IQOS is a heated tobacco product (HTP), composed of an electronic device that heats tobacco sticks called HEETS, and is manufactured by Philip Morris International. IQOS was first launched in Japan in 2014, and is currently sold in more than 70 countries, dominating the HTP global market [1, 2]. The point-of-sale environment is one of the most important but underregulated venues for tobacco marketing [3–5]. Marketing materials and price promotions at points-of-sale increase brand awareness and impulse purchasing [5–8], attract susceptible populations (mainly youth), and serve as smoking cues that might hinder quit attempts and trigger relapse [5, 7–9]. In addition, tobacco products are usually placed at the eye-level, behind the counter, or in a place visible for most, if not all, customers [10–13]. Some tobacco companies pay for dedicated counter spaces or supply the point-of-sale with free display cases [10].

Studies have shown that point-of-sale display and advertisement bans are effective tobacco control measures that can support quit attempts and lead to reduced smoking rates over time [4, 9, 14–16]. Point-of-sale marketing might be especially important for new products like IQOS. The way a new product like IQOS is promoted to retailers at points-of-sale, and their attitude towards it, might affect their direct-to-consumer approach [5].

There is evidence to suggest that tobacco companies might use distinctive marketing strategies to differently target points-of-sale in specific areas, for example; in neighborhoods representing greater proportions of specific ethnic groups [17]. A 2015 systematic review revealed Philip Morris International’s use of an “Integrated Retail Demographic Database Micro-Marketing Tool”, which utilized data gathered from demographic census and retail pricing to customize campaigns and offerings, including price promotions, directed at specific points-of-sale in certain areas [18]. Several studies have found more tobacco marketing and lower cigarette prices in neighborhoods with lower socioeconomic status (SES) and large proportion of ethnic minority individuals [19–22]; however, other studies have documented no clear associations [23, 24]. In the United States, menthol cigarettes marketing was more prevalent in neighborhoods with large proportions of African American residents, while smokeless tobacco was mainly advertised in predominantly White neighborhoods [20].

Israel is a predominantly Jewish country, with the Arab population being its largest ethnic minority group (21.1% of the total population) [25, 26]. More than 80.0% of Arabs reside in all-Arab or majority-Arab localities, 95.0% of which are of low SES [27]. Tobacco and nicotine use is higher among Arabs than Jews (24.4% vs. 19.1% for cigarette; 2.8% vs. 1.2% for IQOS and/or electronic cigarette) [28]. IQOS first entered the Israeli market in 2016 and has been regulated the same as all other tobacco and nicotine products since 2017 [25, 26, 29]. Among other measures, this includes a ban on advertisements in TV, radio, digital media and points-of-sale (2019), a point-of-sale display ban where all tobacco and nicotine products should be concealed at all times (2020), and plain packaging requirements for all of these products (2020) [25, 26, 29].

In a previous Israel-based study [3], concealed point-of-sale audits were carried out at 80 points-of-sale in 2019 and 2020 to assess marketing materials and regulatory compliance *before* the point-of-sale display ban and plain packaging went into effect (January 8 2020) [3]; IQOS/HEETS marketing materials and price promotions were uncommon, but IQOS/HEETS special displays were found at 20% of the audited points-of-sale [3]. However, this study only included a small sample of points-of-sale in Arab neighborhoods (n = 5), and therefore could not compare whether marketing strategies differed between points-of-sale in predominantly Arab vs. Jewish neighborhoods.

This study aims to assess and compare IQOS marketing strategies to and at points-of-sale, regulatory compliance and retailers’ attitudes towards IQOS between points-of-sale in Arab versus Jewish neighborhoods in Israel.

## Methods

### Study design

This manuscript integrates data from two sources:

1. A cross-sectional survey among retailers at points-of-sale that ever-sold IQOS/HEETS (December 2020–April 2021).
2. Concealed point-of-sale audits of retailers that were currently selling IQOS/HEETS (April 2021–July 2021).

For each data collection, points-of-sale in Jewish neighborhoods were randomly selected from the IQOS Israel website, and all points-of-sale in Arab neighborhoods were included in the sample (data from the survey and audits were deidentified and were not matched) [5].

## Cross-sectional survey with retailers

### Settings and procedures

This study examined IQOS marketing strategies at points-of-sale in Israel via a phone survey with owners or managers of points-of-sale that ever-sold IQOS/HEETS in five large cities in Israel: Haifa, Jerusalem, Tel Aviv (all have both Jewish and Arab populations), Beer Sheva (a predominantly Jewish city), and Nazareth (a predominantly Arab city) from December 2020 to April 2021 [5]. We attempted to include points-of-sale from other predominantly Arab cities in Israel (e.g., Um Al-Fahem, Baqa Al-Gharbiah), but these cities had a very minimal number of IQOS points-of-sale (6–8 for each city), and due to funding constraints for RA travel, these points-of-sale were not included.

The initial sample included a total of 713 IQOS points-of-sale (n = 86 in Arab neighborhoods and n = 627 in Jewish neighborhoods) across these cities, which were then contacted by phone to participate in the survey. Of these, only 235 points-of-sale were successfully contacted by phone and 171 were eligible to participate (i.e., ever sold IQOS/HEETS; n = 27 in Arab neighborhoods and n = 144 in Jewish neighborhoods), and of these n = 171, only n = 43 (n = 5 in Arab and n = 38 in Jewish neighborhoods) consented and completed the survey [5]. Thus, additional recruitment efforts included attempting in-person survey data collection by research assistants in a matched sample (by city and SES) of n = 106 points-of-sale that could not be reached by phone (n = 53 in Arab and n = 53 in Jewish neighborhoods). Of these, n = 62 were eligible to participate, with n = 26 consenting and completing the survey (n = 16 in Arab and n = 10 in Jewish neighborhoods).

The final sample included 69 points-of-sale (n = 21 in Arab and n = 48 in Jewish neighborhoods), with an overall response rate of 24.9% (69/277); 35.0% (21/60) in Arab and 27.8% (48/173) in Jewish neighborhoods. Retailers (either point-of-sale manager or owner) who completed the survey were compensated with a 100 New Israeli Shekel online voucher.

### Data collection tool

**Fig. 1:**
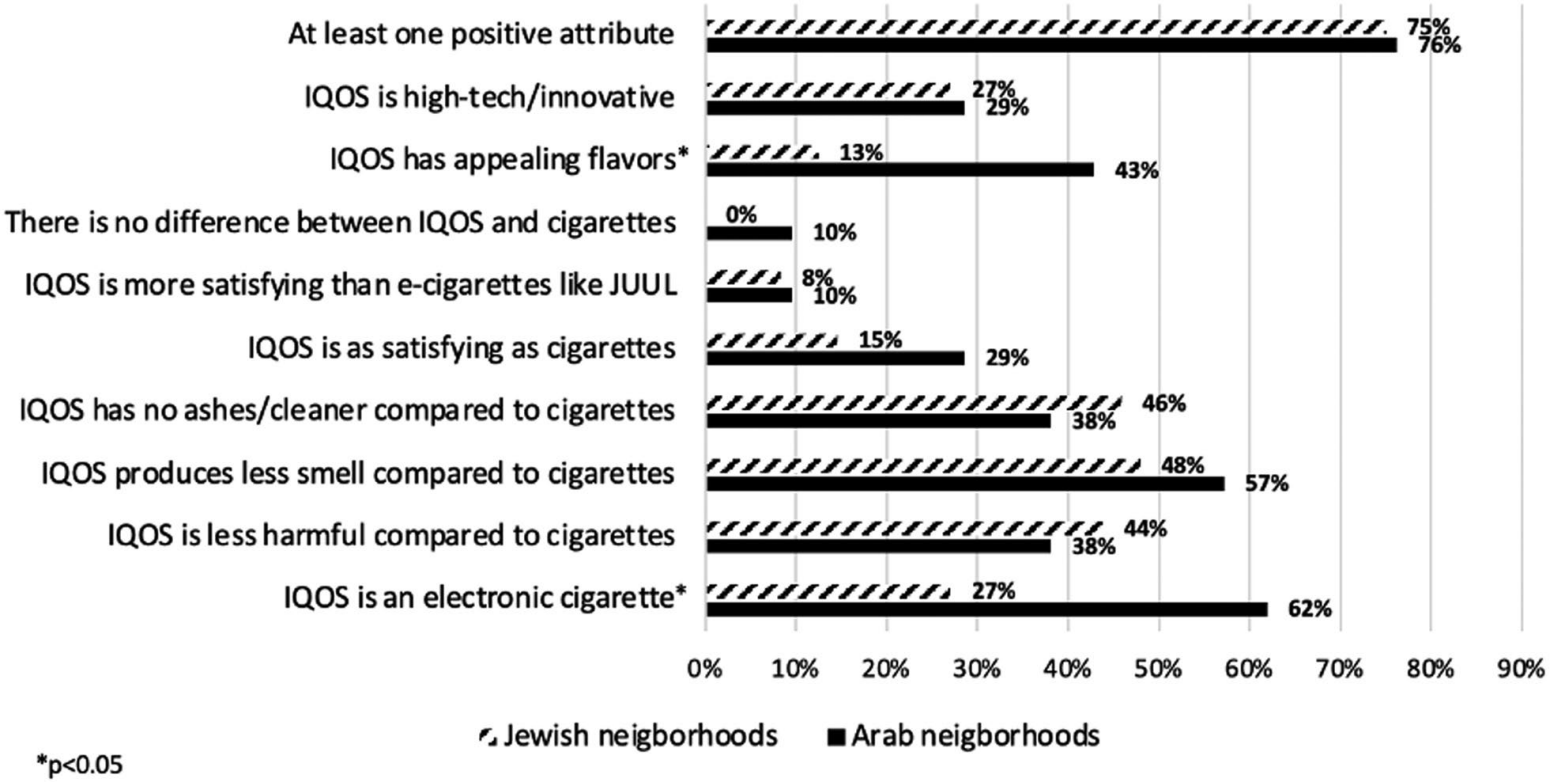
How retailers describe IQOS/HEETS to customers: this shows the percentage of retailers who chose each statement (not mutually conclusive) about how they would describe IQOS/HEETS to their customers who might ask about their tobacco products or IQOS. The start sign denotes statistically significant differences, using Chi-square test (*p* < 0.05)

The survey assessed: (1) Point-of-sale characteristics (i.e., type of store, belonging to a chain, neighborhood SES); (2) participant characteristics (i.e., age, sex, job position at the location, cigarette use status, IQOS use status); (3) number of HEETS flavors sold [< 4, 4–5, or all 6 HEETS flavors], and presence of a special display for IQOS/HEETS; (4) marketing strategies directed at the point-of-sale (e.g., free HEETS samples for the retailer’s personal use, incentives for sales); (5) marketing strategies directed at the customers including promotions (e.g., free HEETS samples for the customers, price discounts), and advertisements (i.e., if the retailer ever promoted IQOS/HEETS online, via social media, via print media, or inside the point-of-sale, recoded into yes/no for each and later recategorized to “any form of ads”), with an option to elaborate on the answers in questions 4 and 5; (6) a question assessing how retailers would communicate with customers about IQOS and/or HEETS (*“How would you describe the IQOS/HEETS to your customers who might ask about your tobacco products or IQOS?”*) with 9 separate check boxes (e.g., *“IQOS is less harmful compared to traditional combustible cigarettes”, “IQOS is as satisfying as cigarettes”,* the full list of options is in Fig. 1); (7) an additional open-ended question asking them to describe their personal opinions of IQOS/HEETS; (8) interactions with a Philip Morris International’s salesperson (the manufacturer of IQOS) such as providing direction on product placement, target market, how to communicate with customers, and providing information about IQOS/HEETS in comparison to other tobacco products (recoded into yes/no for each and with an additional variable of “any interaction”); and (9) Philip Morris International’s reactions to the new tobacco legislation (i.e., retailers were provided education on the new legislation; free cabinets, etc.), with an option to elaborate on the answers in questions 8 and 9.

## Point-of-sale audits

### Settings and procedures

From April to July 2021, trained research assistants conducted concealed, in-person audits among points-of-sale that currently sold IQOS/HEETS in the same large cities (Beer Sheva, Haifa, Jerusalem, Tel Aviv, and Nazareth).

Out of the 195 points-of-sale visited (n = 82 in Arab and n = 112 in Jewish neighborhoods), audits were completed in 129 points-of-sale (n = 48 in Arab and n = 81 in Jewish neighborhoods). Points-of-sale that were not audited were either permanently closed/not found (n = 24; n = 14 in Arab and n = 10 in Jewish neighborhoods), or stopped selling IQOS/HEETS (n = 41; n = 20 in Arab and n = 21 in Jewish neighborhoods). Audits were conducted using a validated surveillance tool, developed based on the Standardized Tobacco Assessment for Retail Settings (STARS), and adapted for IQOS [3].

### Data collection tools

The audit tool assessed: (1) Point-of-sale characteristics (e.g., type of store, belonging to a chain, neighborhood SES); (2) marketing materials inside or outside the point-of-sale such as IQOS/HEETS special display, QR code, or signage for products sold (coded as “any ad” if marketing materials were found for any product); (3) price promotions; (4) visibility of the sold products (sold and visible, sold and not visible, or not sold); (5) prices (i.e., least expensive price of a cigarette pack and HEETS) and any price promotions across products; (6) placement (i.e., within 30 cm of toys or candy, and/or within one meter of the floor); and (7) regulatory compliance (e.g., presence of minimum age signage, presence of a “no smoking” sign, all tobacco and nicotine products in plain packaging, and all tobacco and nicotine products are completely covered and not visible; if a product was visible the point-of-sale was coded as noncompliant).

### Data analysis

Descriptive analysis was conducted using counts and percentages (%) for categorical variables and mean (SD) for continuous variables (e.g., age, product price). Bivariate analyses were conducted using Chi-Square test, Fisher’s exact test or Mann–Whitney test, with Bonferroni correction as appropriate, to assess the differences between points-of-sale in Arab and Jewish neighborhoods. For all analyses, SPSS v27 was used, and a *p* < 0.05 was considered statistically significant. Open coding and thematic analysis were used by the lead researcher (AK) to code and analyze free-text and open-ended questions, which were validated by a senior researcher (YBZ).

## Results

### Cross-sectional survey with retailers

#### Point-of-sale and participant (manager/owner) characteristics

Table 1 summarizes the point-of-sale and participant (manager/owner) characteristics from the retailers’ survey, overall and comparing retailers at Arab vs. Jewish neighborhoods. The surveyed points-of-sale were mainly grocery stores (37.7%, n = 26) or convenience stores not within a gasoline station (34.8%, n = 24). A higher proportion of points-of-sale in Arab neighborhoods were grocery stores (71.4% vs. 22.9%, *p* = *0.001*), and all of them (100.0%) were located in neighborhoods of low- and medium-SES status, compared to 64.6% of points-of-sale in Jewish neighborhoods (*p* = *0.009*).

**Table 1:** Point-of-sale and retailer characteristics, overall and across Arab vs. Jewish neighborhoods

Variable	Total (n = 69) n (%)	Population group		*p* value^†^
		Arab (n = 21) n (%)	Jewish (n = 48) n (%)	
*Point-of-sale characteristics*				
Store type				
Convenience store-no gasoline	24 (34.8)	**4 (19.0)**	**20 (41.7)**	0.001^#^
Convenience store with gasoline	8 (11.6)	**0 (0.0)**	**8 (16.7)**	
Grocery store	26 (37.7)	**15 (71.4)**	**11 (22.9)**	
Liquor store	5 (7.2)	**0 (0.0)**	**5 (10.4)**	
Tobacco store	4 (5.8)	**1 (4.8)**	**3 (6.2)**	
Other (café, kiosk)	2 (2.9)	**1 (4.8)**	**1 (2.1)**	
Neighborhood socioeconomic status (SES)				
Non-residential	7 (10.1)	**0 (0.0)**	**7 (14.6)**	0.009^#^
Low	10 (14.5)	**5 (23.8)**	**5 (10.4)**	
Medium	42 (60.9)	**16 (76.2)**	**26 (54.2)**	
High	10 (14.5)	**0 (0.0)**	**10 (20.8)**	
*Participants characteristics*				
Age, mean (SD)	39.1 (9.9)	39.4 (9.5)	38.9 (10.1)	0.806^^^
Sex				
Male	62 (89.9)	17 (81.0)	45 (93.8)	0.188^#^
Female	7 (10.1)	4 (19.0)	3 (6.2)	
Position at the point-of-sale				
Owner	16 (23.2)	4 (19.0)	12 (25.0)	0.760^#^
Manager	53 (76.8)	17 (81.0)	36 (75.0)	
Cigarette smoking status*				
Current smoker	26 (38.2)	7 (33.3)	19 (40.4)	0.833
Past smoker	14 (20.6)	5 (23.8)	9 (19.2)	
Never smoker	28 (41.2)	9 (42.9)	19 (40.4)	
IQOS use status*				
Current user	8 (11.8)	1 (4.8)	7 (14.9)	0.163^#^
Past user	9 (13.2)	5 (23.8)	4 (8.5)	
Never user	51 (75.0)	15 (71.4)	36 (76.6)	

^†^Chi square test, unless stated otherwise. ^#^Fishers exact test. ^^^Mann–Whitney test. *Missing: **Participants characteristics:** Cigarette smoking n = 1; IQOS use status n = 1 (both are retailers in Jewish neighborhoods). **Bold** indicates between-group statistically significant differences (Bonferroni correction)

#### IQOS marketing strategies

Table 2 summarizes IQOS/HEETS marketing strategies overall, and compares retailers in Arab vs. Jewish neighborhoods. Compared to Jewish neighborhoods, a higher proportion in Arab neighborhoods carried less than 4 HEETS flavors (66.7% vs. 17.4%, *p* < *0.001*) and less IQOS special displays (25.0% vs. 53.2%, *p* = *0.034*) (Table 2).

**Table 2:** Point-of-sale IQOS/HEETS marketing strategies, overall and across Arab vs. Jewish neighborhoods

Variable	Total (n = 69) n (%)	Population group		*p* value^†^
		Arab (n = 21) n (%)	Jewish (n = 48) n (%)	
Number of HEETS flavors*				
< 4	22 (32.8)	**14 (66.7)**	**8 (17.4)**	< 0.001
4–5	20 (29.9)	5 (23.8)	15 (32.6)	
All 6	25 (37.3)	2 (9.5)	23 (50.0)	
Special display*	30 (44.8)	5 (25.0)	25 (53.2)	0.034
Promotions to points-of-sale*				
Free HEETS samples	13 (21.3)	4 (20.0)	9 (22.0)	1.000
Price discounts for the retailer’s own purchase of HEETS/IQOS	18 (29.5)	8 (42.1)	10 (23.8)	0.174
Paraphernalia	7 (10.6)	6 (30.0)	1 (2.2)	0.002^#^
Other gifts^§^	6 (9.7)	4 (20.0)	2 (4.8)	0.079^#^
Price discounts, rebates, or incentives based on promoting their products	27 (41.5)	7 (36.8)	20 (43.5)	0.621
Incentives for sales of their products	22 (33.8)	5 (25.0)	17 (37.8)	0.315
Invitations to IQOS parties or events	20 (29.9)	10 (47.6)	10 (21.7)	0.032
Promotions to customers*				
Free HEETS samples	4 (6.7)	1 (6.3)	3 (6.8)	1.000^#^
Other gifts^‡^	5 (8.3)	3 (18.8)	2 (4.5)	0.112^#^
Price promotions^$^	7 (11.9)	1 (7.1)	6 (13.3)	1.000^#^
Price discounts	23 (37.7)	3 (18.8)	20 (44.4)	0.069
Coupons	6 (10.5)	1 (6.7)	5 (11.9)	1.000^#^
Special prices for members	4 (6.9)	2 (12.5)	2 (4.8)	0.303^#^
Advertisements by the retailer*				
Any form of ads	38 (58.5)	10 (50.0)	28 (62.2)	0.356
Online	5 (7.7)	0 (0.0)	5 (11.1)	0.313^#^
Social media	3 (4.8)	1 (5.0)	2 (4.7)	1.000^#^
Print media	2 (3.1)	1 (5.0)	1 (2.3)	0.531^#^
Inside the point-of-sale	37 (58.7)	10 (50.0)	27 (62.8)	0.337

^†^Chi square test, unless stated otherwise. ^#^Fishers exact test. *Missing: **Number of HEETS flavors** n = 2; **Special display** n = 2; **Promotions to points-of-sale**: free HEETS samples n = 8; price discounts for your own purchases n = 8; paraphernalia n = 3; other gifts n = 7; price discounts, rebates, or incentives based on promoting their products n = 4; incentives for sales n = 4; invitations to parties n = 2; **Promotions to costumers:** free HEETS samples n = 9; paraphernalia is zero for all points-of-sale; other gifts n = 9; price promotions n = 10; price discounts n = 8; coupons n = 12; special prices for members n = 11; Special discounts for military/students was zero for all points-of-sale; **Advertisements**: any form of ad n = 4 (if all items were missing); online n = 4; social media n = 6; print media n = 5; inside the point-of-sale n = 6. ^§^Other gifts given to retailers included lighters, lighter stands, and entering a draw for flights abroad. ^‡^Other gifts given to customers included lighters. ^$^Price promotions offered to customers such as buy one get one free, 2 NIS off the price of HEETS, or buy IQOS and get a free HEETS package. **Bold** indicates between-group statistically significant differences (Bonferroni correction)

More retailers in Arab versus Jewish neighborhoods received invitations to IQOS events/parties (47.6% vs. 21.7%, *p* = *0.032*) and paraphernalia (30.0% vs. 2.2%, *p* = *0.002*). The most prevalent form of promotions targeting customers were price discounts (18.8% and 44.4% of points-of-sale in Arab and Jewish neighborhoods, respectively, *p* = 0.069) (Table 2). Only retailers in Arab neighborhoods mentioned receiving lighter stands (n = 2), entering a draw to win flights abroad (n = 1), and receiving points when referring customers (n = 1). More than half of all points-of-sale carried IQOS promotional materials (Table 2); these were mainly electronic and/or non-electronic signs that said “here you can buy heated tobacco products” or “heated tobacco units”, small flags that say the same or advertise a price promotion, and/or special display cases for IQOS/HEETS. A few retailers mentioned that Philip Morris International sent saleswomen to set up a small stand and promote IQOS directly to customers (6 in Arab neighborhoods and 3 in Jewish neighborhoods).

#### Open-ended questions

Retailers from points-of-sale in Arab neighborhoods emphasized their part in promoting the product to their customers and connecting the customers with sales representatives. For example, one participant used her personal experience as a promotional strategy: *“When people come to my shop and see me use it, they get curious and start asking me about it, I tell them about my personal experience and how I used to smoke Marlboro but when I switched to IQOS I stopped coughing in the morning and it doesn't stink your clothes or furniture”*.

Another participant stated that the point-of-sale was acting as a “middle man” by connecting the customer with a Philip Morris International’s sales representative: *“the shop was the intermediary; the company's representative asked us to connect him with the customers if anyone asks about IQOS or was interested in trying it”,* and mentioned collecting ID numbers and phone numbers to register customers for a user database, and received points for each person. Others referred to the representative’s direct interaction with customers; *“the representative and I try to tell customers about IQOS, that it can meet their requirements and is less harmful and smoke-free”.*

#### Retailers’ attitudes towards IQOS and interactions with a Philip Morris International’s representatives

More retailers from points-of-sale in Arab neighborhoods stated that IQOS is an electronic cigarette (61.9% vs. 27.1%, *p* = *0.006*) and found its flavors to be appealing (42.9% vs. 12.5%, *p* = *0.009*) (Fig. 1: how retailers describe IQOS/HEETS to customers). Overall, 42.0% of retailers stated that IQOS is less harmful compared to cigarettes (43.8% and 38.1% among retailers from points-of-sale in Jewish and Arab neighborhoods, respectively).

Overall, 51 respondents answered the open-ended question describing their opinion of IQOS/HEETS, 36 of which had a favorable attitude towards the product, with “less harmful/toxic” being the descriptor used the most (n = 12/36; n = 4/10 Arab and n = 6/26 Jewish). One Arab retailer stated that “*this product doesn't harm the user so even if I use up 3 packets of HEETS a day it doesn't harm me*”, a Jewish retailer referred to the US Food and Drug Administration (FDA) in his answer; “*the fact that IQOS is approved by the FDA proves that it is a quality product and not harmful to health*.”

Table 3 lists the retailers’ interactions with a Philip Morris International’s representative and Philip Morris International’s reaction to the point-of-sale display ban. More retailers from points-of-sale in Arab than Jewish neighborhoods reported having any form of interaction with a Philip Morris International’s representative (80.0% vs. 51.2%, *p* = *0.029*) with no statistically significant differences in regards to the detailed nature of those interactions.

**Table 3:** Interactions with a Philip Morris International representative and Philip Morris International’s reaction to the point-of-sale display ban, overall and across points-of-sale in Arab vs. Jewish neighborhoods

Variable	Total (n = 69) n (%)	Population group		*p* value^†^
		Arab (n = 21) n (%)	Jewish (n = 48) n (%)	
Specific IQOS/HEETS salesperson*	25 (37.9)	6 (28.6)	19 (42.2)	0.287
Interaction with Philip Morris International salesperson*				
Any interaction	38 (60.3)	16 (80.0)	22 (51.2)	0.029
Provided direction on placement	32 (50.0)	13 (65.0)	19 (43.2)	0.106
Provided information on the target market	16 (25.8)	2 (10.0)	14 (33.3)	0.050
Provided direction how to communicate with consumers	21 (35.0)	7 (35.0)	14 (35.0)	1.000
Provided information on IQOS/HEETS versus other tobacco products	31 (51.7)	13 (65.0)	18 (45.0)	0.144
Philip Morris International’s reaction to the point-of-sale display ban				
Any interference*	51 (81.0)	17 (89.5)	34 (77.3)	0.318
Provided education regarding the tobacco legislation	26 (37.7)	11 (57.9)	15 (34.1)	0.096
Advised on how to work around the tobacco legislation	8 (11.6)	5 (26.3)	3 (6.8)	0.050^#^
Provided free cabinets, display cases and/or signage to address the tobacco legislation	36 (52.2)	12 (63.2)	24 (54.5)	0.585
Changed their promotional strategies for products such as electronic cigarettes and/or IQOS/HEETS	7 (10.1)	3 (15.8)	4 (9.1)	0.667^#^
Minimized the importance of compliance with the tobacco legislation	4 (5.8)	1 (5.3)	3 (6.8)	1.000^#^
Sold cabinets, display cases and/or signage to address the tobacco legislation	8 (11.6)	0 (0.0)	8 (18.2)	0.095^#^

^$^Includes ticking at least one statement from a-g. ^†^Chi square test, unless stated otherwise ^#^Fisher's exact test. *Missing: Specific IQOS/HEETS salesperson n = 3; **Interaction with a Philip Morris International salesperson:** Any interaction n = 6; provide direction on placement n = 5; target market n = 7; communicate with consumers n = 9; information on product n = 9; **Philip Morris International’s reaction to the point-of-sale display ban:** All items n = 6

Philip Morris International’s representatives assisted the majority of points-of-sale implement the display ban (89.5% in Arab and 77.3% in Jewish neighborhoods), the only borderline significant difference was more points-of-sale in Arab neighborhoods being advised on how to navigate and overcome regulatory restrictions, (26.3% vs. 6.8%, *p* = *0.05*) (Table 3). This included, for example, being given instructions on how to arrange the products behind the cover to make it easier to access and sell them, being directly informed about new campaigns and promotions, and repeatedly given information about the products.

### Point-of-sale audits

#### Point-of-sale characteristics

Table 4 summarizes point-of-sale characteristics, marketing material, placement, promotion and regulatory compliance data from all audited points-of-sale, and across Arab vs. Jewish neighborhoods. The audited points-of-sale were mainly convenience stores not within a gasoline station (45.0%, n = 58) or convenience stores within a gasoline station (31.0%, n = 40). Significantly more points-of-sale in Arab neighborhoods were located in areas of low SES (75.0%, n = 36), compared to only 2.4% (n = 2) of points-of-sale in Jewish neighborhoods (*p* < *0.001*) (Table 4).

**Table 4:** Point-of-sale characteristics, marketing material, placement, promotions and regulatory compliance, overall and across Arab vs. Jewish neighborhoods

Variable	Total (n = 129) n (%)	Population group		*p* value^†^
		Arab (n = 48) n (%)	Jewish (n = 81) n (%)	
Point-of-sale characteristics				
*Type of store*				
Convenience store with gasoline	40 (31.0)	11 (22.9)	29 (35.8)	0.440
Convenience store-no gasoline	58 (45.0)	23 (47.9)	35 (43.2)	
Grocery store/ supermarket	21 (16.2)	9 (18.8)	12 (14.8)	
Other ^Ÿ^	10 (7.8)	5 (10.4)	5 (6.2)	
*Neighborhood socioeconomic status (SES)*				< 0.001
Non-residential	17 (13.2)	4 (8.3)	13 (15.9)	
Low	37 (28.7)	**36 (75.0)**	**2 (2.4)**	
Medium	56 (43.4)	**7 (14.6)**	**49 (59.8)**	
High	19 (14.7)	**1 (2.1)**	**18 (21.9)**	
Marketing materials (any)	103 (79.8)	35 (72.9)	68 (82.9)	0.131
*IQOS/HEETS*				
Any ad/sign^*¶*^	59 (57.3)	20 (57.1)	39 (57.4)	0.475
Special display^‡^	39 (66.1)	11 (55.0)	28 (71.8)	0.142
Brand colors^‡^	45 (76.3)	14 (70.0)	31 (79.5)	0.111
*Cigarettes*				
Any ad/sign^*¶*^	84 (81.6)	26 (74.3)	58 (85.3)	0.162
Brand names*	25 (29.8)	12 (46.2)	13 (22.4)	0.024
Price stickers*	60 (71.4)	17 (65.4)	43 (74.1)	0.044
Visibility**				
IQOS	16 (76.2)	4 (66.7)	11 (68.8)	1.000^#^
HEETS	70 (54.3)	19 (39.6)	51 (62.2)	0.010
Cigarettes	81 (62.8)	27 (56.3)	54 (65.9)	0.237
HEETS flavors; M (SD)	4.2 (1.8)	3.5 (1.9)	4.6 (1.6)	0.001^^^
IQOS/HEETS placement				
Within 30 cm of toys or candy	20 (15.5)	5 (10.4)	15 (18.3)	0.219
Within 1 m of the floor	10 (7.8)	0 (0.0)	10 (12.2)	0.013^#^
Price promotion				
IQOS/HEETS	14 (10.9)	4 (8.3)	10 (12.2)	0.479
Other tobacco product	33 (25.6)	9 (18.8)	24 (29.3)	0.171
Prices; M (SD)				
HEETS	30.2 (1.7)	29.5 (1.2)	30.7 (1.9)	< 0.001^^^
Cheapest Philip Morris International cigarette	25.4 (2.1)	24.5 (1.5)	26.1 (2.3)	< 0.001^^^
Most expensive Philip Morris International cigarette	38.5 (3.2)	38.4 (1.4)	38.9 (4.0)	0.004^^^
Regulatory compliance				
Minimum age signage	75 (58.1)	23 (47.9)	52 (63.4)	0.070
No smoking sign	37 (28.7)	18 (37.5)	19 (23.2)	0.088
Plain packaging	95 (73.6)	30 (62.5)	65 (79.3)	0.027
Display ban^&^	19 (15.1)	12 (25.5)	7 (8.8)	0.011

^†^Chi square test, unless stated otherwise. ^#^Fisher’s exact test. ^^^Mann–Whitney test. ^Ÿ^Other: Liquor store n = 1; Tobacco shop n = 3; Coffee shop n = 3; Candy store n = 2; Spice shop n = 1. ^¶^Out of those with any internal ad (n = 103; Arab n = 35 and Jewish n = 68). ^‡^Out of those with any *IQOS* ads (n = 59; Arab n = 20 and Jewish n = 39). *Out of those with any *cigarette* ads (n = 84; Arab n = 26 and Jewish n = 58). **Not sold: IQOS device n = 108 (n = 42 Arab and n = 66 Jewish). ^&^Excluding n = 3 tobacco shops (Arab n = 1 and Jewish n = 2) that the display ban does not apply to. **Bold** indicates between-group statistically significant differences (Bonferroni correction)

#### Marketing materials, prices and price promotions

**Fig. 2:**
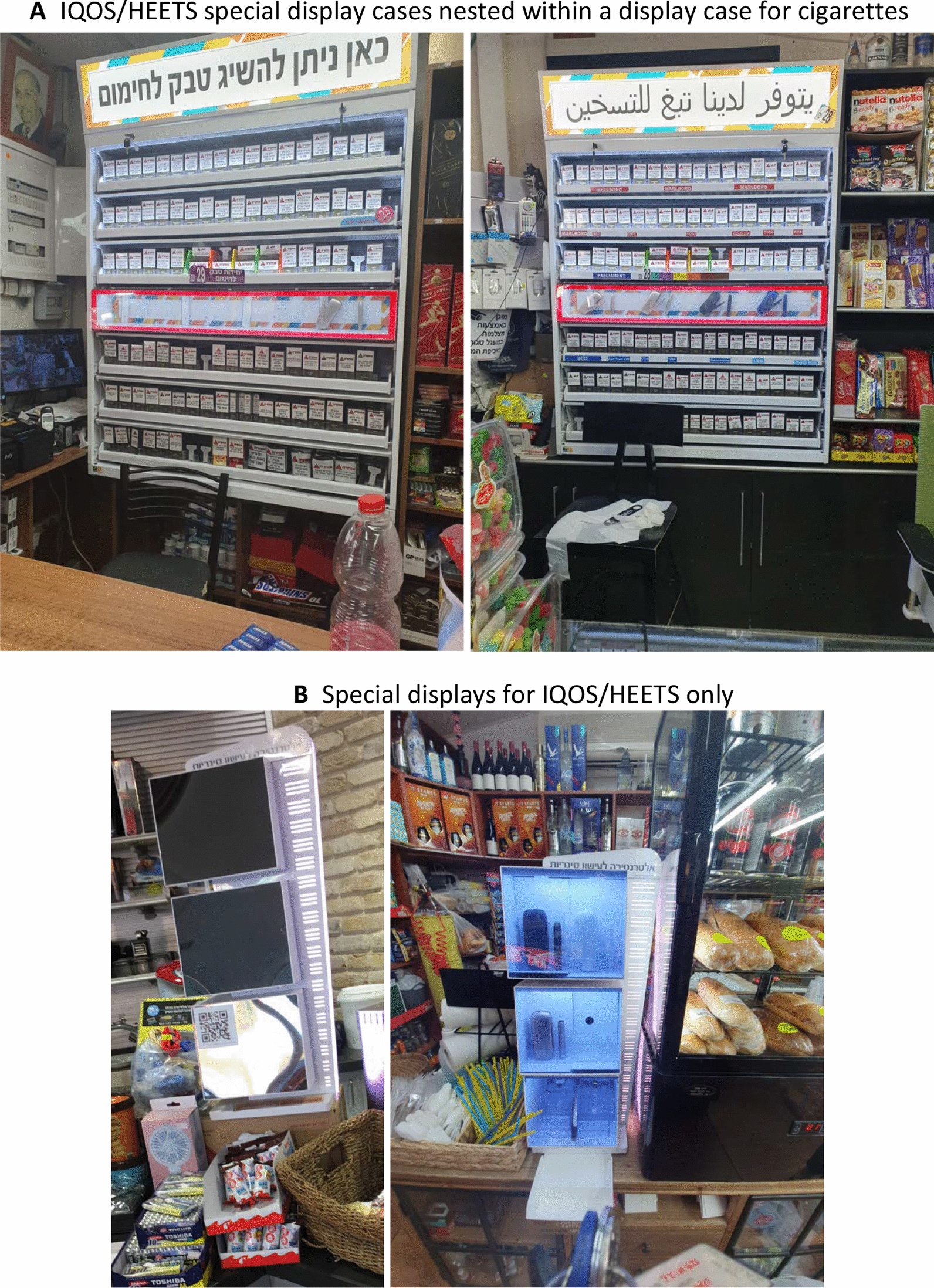
**A** IQOS/HEETS special display cases nested within a display case for cigarettes: On the left: IQOS special display with the Hebrew words for “here you can buy heated tobacco”. On the right: IQOS special display with the Arabic words for “we have heated tobacco”. Both displays use IQOS’ brand colors, sell the IQOS device, are non-compliant with the display ban, have a special placement for HEETS in the colored section on top of the where the device is displayed with HEETS price stickers. The picture on the left also contains tobacco products not in plain packaging. Both are from points-of-sale in Arab neighborhoods. **B** Special displays for only IQOS/HEETS: IQOS special display cases with the Hebrew words for “alternative for smoking cigarettes”. The one on the left is with the light turned off and has a QR code, and the one on the right is with the light turned on. Both are found at points-of-sale in Jewish neighborhoods

The vast majority of points-of-sale (79.8%, n = 103) had internal and/or external ads for any tobacco or nicotine product (72.9% Arab, n = 35 and 82.9% Jewish, n = 68); of which more than half was IQOS-indirect internal signage (57.1% Arab, n = 20/35 and 57.4% Jewish, n = 39/68), such as signs that said “heated tobacco units”, or “here you can buy heated tobacco”. The majority of points-of-sale that had any IQOS/HEETS signage included HEETS brand colors (70.0% Arab, n = 14/20 and 79.5% Jewish, n = 31/39), and IQOS/HEETS special displays (55.0% Arab, n = 11/20 and 71.8% Jewish, n = 28/39) (Table 4). Some of the special display cases provided by Philip Morris International were “discreet”; they had a light switch that makes the product visible only when turned on, and a few of them also featured a sticker with a QR code (Fig. 2A, B: IQOS special displays at points-pf-sale in Arab and Jewish neighborhoods, respectively).

The majority of points-of-sale in Arab (74.3%, n = 26/35) and Jewish neighborhoods (85.3%, n = 58/68) had cigarettes-specific internal signage, such as signs that said “cigarettes”. In contrast, significantly more points-of-sale in Arab neighborhoods mentioned a specific cigarette brand name (46.2%, n = 12/26 vs. 22.4% in Jewish neighborhoods, n = 13/58; *p* = *0.024*).

On average, points-of-sale in Arab neighborhoods carried fewer HEETS flavors (3.5 vs. 4.6, *p* = *0.001*), and sold them at a lower price (29.5 NIS vs. 30.7 NIS, *p* < *0.001*). Similarly, points-of-sale in Arab neighborhoods sold Philip Morris International cigarettes at a lower price on average (cheapest Philip Morris International cigarette: 24.5 NIS vs. 26.1 NIS, *p* < *0.001*; most expensive Philip Morris International cigarette: 38.4 NIS vs. 38.9 NIS, *p* = *0.004*). Price stickers that either indicated the advertised price or a price promotion were found at significantly more points-of-sale in Jewish neighborhoods (74.1%, n = 43/58 vs. 65.4% in Arab neighborhoods, n = 17/26; *p* = *0.044*).

#### Placement, visibility and regulatory compliance

IQOS/HEETS were placed within 1 m of the floor only in points-of-sale in Jewish neighborhoods (12.2%, n = 10). IQOS was highly visible in the points-of-sale that sold it (68.8% in Jewish and 66.7% in Arab neighborhoods), but the visibility of HEETS was higher among points-of-sale in Jewish neighborhoods (62.2% vs. 39.6% in Arab neighborhoods, *p* = *0.010*).

A significantly higher proportion of points-of-sale in Arab neighborhoods had products in their original packaging (i.e., not in plain packaging as required by law) compared to Jewish neighborhoods’ points-of-sale (37.5% vs. 20.7%, *p* = *0.027*), but a higher proportion were compliant with the display ban (25.5% vs. 8.8%, *p* = *0.011*).

## Discussion

Findings from these studies show that, in general, Philip Morris International employed similar marketing strategies at points-of-sale in Arab and Jewish neighborhoods, without clear specific targeting. Nonetheless, a more personalized marketing approach (personal communication, social events) was more prevalent at points-of-sale in Arab neighborhoods.

Our findings of no over-targeting of points-of-sale in Arab neighborhoods compared to Jewish ones is in alignment with our previous study that explored Philip Morris International’s marketing in print media across population groups and media outlets in Israel [25, 26, 29]. However, this is contradictory to research in other countries, where clear targeting of minority populations was reported [19–22]. This might be attributed to IQOS being a relatively new product in the market, which would warrant a focus on the majority population in order to increase market share.

The use of different marketing strategies for points-of-sale in Arab or Jewish neighborhoods, such as more personal communication with retailers and invitation to social events could be attributed to a form of close network marketing that might be due to cultural differences. It has been suggested that in the Arab population, business relationships might be heavily influenced by personal ties [30]. In addition, this might reflect the presence of different marketing teams (Arab and Jewish) to carry out in-person communications and outreach activities.

The majority of surveyed retailers had a positive attitude towards IQOS/HEETS. These perceptions are of great importance because they might influence how retailers communicate with customers and could be the result of Philip Morris International’s marketing efforts directed at the points-of-sale. While some retailers talked about themselves becoming salespeople or intermediaries who connected potential customers with a Philip Morris International representative, retailers in Arab neighborhoods were more inclined to use their personal experiences using IQOS to increase their credibility when promoting the product to customers, even though more retailers in Jewish neighborhoods were current IQOS users. The majority of points-of-sale in Arab neighborhoods were small businesses (i.e., grocery stores; 71.4%), owned and operated by community members who can promote IQOS sales by influencing their customers through personal relationships, cultural cohesion, and embedded trust [30].

Our findings suggest that points-of-sale were used by Philip Morris International as a tool to directly market IQOS to consumers, by sending saleswomen to set up small IQOS stands inside the points-of-sale, and allowing the representative the freedom to talk to customers in order to promote the product, both of which were more prevalent at points-of-sale in Arab neighborhoods. The use of saleswomen is a newer form of what was known in the past as “cigarette girls”, which was previously used by Philip Morris International’s branch in Australia in 2000 [31].

Philip Morris International’s active involvement in helping retailers implement the display ban might have also contributed to the retailers’ positive perception of IQOS, especially with signage that serve as constant reminders of the product. Research from Scotland also highlighted the tobacco industry’s role in helping points-of-sale implement display bans and how to work around them [32]. A survey by The Israel Democracy Institute in 2021 indicated that Arab residents expressed lower trust in the local authorities (32.0% vs. 62.0% of Jewish residents) [33], which might create an opportunity for external entities to influence the retailers by offering guidance, such as advising on how to work around the display ban.

Internal IQOS signage was found in more than half the audited points-of-sale in Arab and Jewish neighborhoods. However, these were mostly indirect and did not explicitly state the brand name, but used more general statements (such as “heated tobacco”). Nonetheless, a high proportion of the signs (76.3%) used specific colors that correspond to HEETS flavors which could be interpreted as branded advertisement, and therefore forbidden by law. Additionally, we found that some retailers were provided with display cases that only show the product when a switch is turned on, thereby subtly violating the display ban. These findings strengthen previous results showing the various ways in which Philip Morris International circumvents legislation [3, 34].

### Limitations

These two studies used cross-sectional data that might not be representative of all points-of-sale in Israel, especially in the Arab population. We only assessed points-of-sale in major cities, and our data collection efforts resulted in a small sample size for both studies. Philip Morris International might have employed other marketing tactics in other cities, or in more regional and rural areas. However, the IQOS website listed very few IQOS points-of-sale located in other dominant Arab cities or more regional and rural localities. Additionally, we collected data both via phone, online, and face-to-face, with differences in data collection between Arab and Jewish points-of-sale (Jewish points-of-sale: 79.2% by phone/online [n = 38/48], Arab: 76.2% face-to-face [n = 16/21]), which might have impacted our results.

The majority of points-of-sale in Jewish neighborhoods were of middle- and high-SES, while the points-of-sale in Arab neighborhoods were of either medium- or low-SES, suggesting that differences might be based on economic, rather than ethnic factors, which cannot be differentiated within the scope of these studies. In addition, there might be some differences based on other factors, such as the city, including predominant population (mixed, predominant Arab, predominant Jewish), and store type. The small sample size precluded us from running more analyses to adjust for these factors. The data for both studies was collected at different points in time, which might have impacted the results. However, there were no differences in legislation or implementation during these times.

Nazareth was the only majority-Arab city included in these studies, and it has both a Christian and Muslim population which could have affected the results and might also limit the representativeness of our sample. Currently, there is no available data on differences in smoking rates between Muslims and Christians, but a study conducted in 2012 showed that Muslim Arabs had a higher secondhand smoking exposure at home (55.4%), compared to 49.0% of Christian Arabs [35]. This could be attributed to differences in smoking behaviors in these two subpopulations. As mentioned previously, the IQOS website had a very small number of points-of-sale in other dominantly Arab cities with a higher proportion of Muslim residents (e.g., Um Al-Fahem).

### Policy implications

Our findings point to the many strategies covertly employed by the tobacco industry to circumvent the point-of-sale advertisement and display bans, including the use of indirect marketing through colors, generic signage and QR codes. These findings highlight the need for more specific wording of the legislation to prevent all advertisement at the point-of-sale. Instead of using ‘general’ wording as currently mentioned in the advertisement ban legislation, the government should consider using specific wording to describe what is allowed and what is not allowed. For example, governments could specify the exact size, colors, font and wording allowed on generic signs, similar to what was done in Australia and Scotland [36, 37].

The interaction between representatives of the tobacco industry and retailers should be limited and specified in the legislation, including banning tobacco companies from holding social events or providing gifts or merchandize to the retailers. Moreover, a clear enforcement plan should be established and followed to ensure compliance by both the tobacco companies and retailers, including clear communicating of all regulatory changes to all parties involved, training, and specific inspectors to oversee compliance and impose fines if needed.

## Conclusions

Our results indicate a high level of regulatory non-compliance and legislation circumvention in both Arab and Jewish populations, with some tailoring of marketing strategies to the Arab sector. Results stress the need for continuous surveillance and regulatory enforcement with more precise legislation specifying exactly what is allowed or not. Banning direct interaction between tobacco companies and retailers might help reduce tobacco industry interference, and contribute to reducing smoking-related health disparities.

## Data Availability

The datasets used and/or analyzed during the current study are available from the corresponding author on reasonable request.

## Abbreviations

FDA: Food and Drug Administration
HTTP: Heated tobacco product
SD: Standard deviation
SES: Socioeconomic status
STARS: Standardized Tobacco Assessment for Retail Settings
